# Effects of *KRTAP20-1* Gene Variation on Wool Traits in Chinese Tan Sheep

**DOI:** 10.3390/genes15081060

**Published:** 2024-08-12

**Authors:** Lingrong Bai, Huitong Zhou, Jinzhong Tao, Jon G. H. Hickford

**Affiliations:** 1 International Wool Research Institute, Faculty of Animal Science and Technology, Gansu Agricultural University, Lanzhou 730070, China; lingrong.bai@lincolnuni.ac.nz (L.B.); huitong.zhou@lincoln.ac.nz (H.Z.); tao_jz@nxu.edu.cn (J.T.); 2 Gene-Marker Laboratory, Faculty of Agriculture and Life Sciences, Lincoln University, Lincoln 7647, New Zealand; 3 College of Animal Science and Technology, Ningxia University, Yinchuan 750021, China

**Keywords:** keratin-associated protein 20-1 (KAP20-1), mean fibre curvature (MFC), fine wool fibre, heterotypic hair fibre

## Abstract

Chinese Tan sheep lambs are recognised for having tight ‘spring-like’ curly wool when young, but this phenotype disappears with age. This wool consists of shorter, fine wool fibres (which are usually unmedullated) and heterotypic hair fibres (which are frequently medullated), which are referred to as ‘halo hair’. Both the wool and hair fibres consist of α-keratin proteins embedded in a keratin-associated protein (KAP) matrix. Of these KAPs, the KAP20-1 gene (designated *KRTAP20-1*) and its effect on four fibre traits (mean fibre curvature, mean fibre diameter, fibre diameter standard deviation, and coefficient of variation of fibre diameter) of Tan lambs was studied. Seven previously identified *KRTAP20-1* variants (*A*, *B*, *D*, *E*, *F*, *G*, and *H*) of *KRTAP20-1* were revealed, but the previously identified variant *C* was not present. Of the seven variants detected, only two (*A* and *G*) were common and present at frequencies greater than 5%, and the effect of these on the fibre traits of the finer wool fibres was assessed. It was found that variant *G* was associated with an increased mean fibre curvature in these wool fibres. This suggests that *KRTAP20-1* might possibly be expressed differentially in the two fibre types, which may be of future value in breeding.

## 1. Introduction

Wool fibres are mainly composed of proteins. There are two types of proteins: α-keratins and keratin-associated proteins (KAPs). The keratins are built into structures called keratin intermediate filaments (KIFs), and the KAPs function as a matrix-like substrate that crosslinks these KIFs [1]. The KAPs appear to play a role in wool fibre structure, and some have been revealed to be associated with variation in various fibre traits across different breeds of sheep [2,3,4,5] and goats [6,7,8]. In humans, eighty-nine KAP genes (*KRTAPs*) that are thought to be functional have been detected [9,10,11], but the study of KAP genes in sheep is less developed.

The KAPs are classified into three groups: the high sulphur (HS) proteins, the ultrahigh sulphur (UHS) proteins, and the high glycine-tyrosine (HGT) proteins, and they form a complex matrix that crosslinks and embeds the KIFs [12]. Understanding the genes encoding the KAPs and wool keratins is probably crucial to understanding the genetic factors that determine the characteristics of the wool fibre, so these genes (*KRTAPs* and *KRTs*, respectively) represent key targets for further candidate gene association studies.

The lambs of the Tan sheep of northwestern China are known for producing tight ‘spring-like’ white curly fleeces. After processing, the lamb pelts with curly fleeces are lightweight, which makes them well suited to producing furniture coverings, carpets, fur coats, and various forms of handicraft. However, the ‘spring-like’ curly or halo fibres only grow for an average of 35 days following birth (called the ‘Er-mao’ period), and after this period, the curl pattern gradually disappears. The mechanisms behind this change are still unclear.

Previous studies of wool fibre have revealed that *KRTAPs* can influence the curvature, growth rate, and uniformity of Tan sheep fibre. For example, variation in *KRTAP6-1* has been related to the length of straightened fibres, number of curls per unit of distance along the fibre, and the degree of fibre curling at birth and at Er-mao [13]. Variation in *KRTAP8-1* can affected the consistency of the diameter of Tan sheep wool fibres as described by the coefficient of variation in fibre diameter (CVFD) [3], and variation in a related gene, *KRTAP8-2*, has been associated with the length of straightened fibres and the degree of crimp of fibres in the Er-mao period [14].

An expression study [15] revealed that the genes for keratins K5, K14, K25, K27, K31, K33 (now K33A), K33B, K34, K71, K83, and K2.11 (now K86) and keratin-associated proteins KAP1-1, KAP2-3, and KAP3-2 were highly expressed in the skin of Tan sheep; only the gene for KAP13-1 was differentially expressed between one-month-old and four-year-old Tan sheep.

In Merino-cross sheep, *KRTAP20-1* variation has been reported to be associated with wool yield and variation in mean fibre diameter (MFD) [16], but it is not known whether this effect is observed in sheep of a different breed, such as Tan sheep. This study was therefore designed to ascertain whether *KRTAP20-1* variation exists in Tan sheep, whether it affects Tan lamb fibre traits, and if it specifically affects the characteristics of the fine wool fibres versus the heterotypic hair fibres.

## 2. Materials and Methods

### 2.1. The Tan Sheep Investigated and Wool Trait Measurements

Two hundred and thirty-three lambs of the Chinese Tan breed were studied, these being the offspring of ten sires. Most of the lambs were singletons at birth, but there were six (three pairs) of twins. To avoid potentially confounding subsequent analyses, the twins were eliminated from the analyses, leaving 227 single lambs.

Fibre samples were gathered from the mid-side region of the lambs near the middle of the Er-mao period (at day 35 postpartum). For each sample, the finer wool fibres were manually separated from the heterotypic hair fibres based on their difference in fibre length and diameter. This was achieved by placing each sample on a flannel board and using a rigid card in one hand to press the bottom of all the fibres onto the board. The other hand was then used to pull and remove the longer hair fibres, with the process being iterated a number of times to make sure that the fine wool fibres and heterotypic hair fibres were all separated.

The mean fibre curvature (MFC), MFD, fibre diameter standard deviation (FDSD), and CVFD of the fine wool fibres and hair were then measured. The fine wool samples were assessed by Pastoral Measurements Limited (Timaru, New Zealand), while the hair samples were measured by the New Zealand Wool Testing Authority (NZWTA—Ahuriri, Napier, New Zealand), using International Wool Textile Organisation (IWTO) standardized testing methods.

Venous blood samples from each of the lambs were collected onto TFN paper (Munktell Filter AB, Falun, Sweden). These samples were air-dried and stored in the absence of light at ambient temperature until needed for further analysis. The genomic DNA which had bound to the paper was prepared for PCR amplification as follows.

To purify the genomic DNA attached to the TFN paper, a 1.2 mm disk was punched from the blood spot on the card and placed in a 0.7 mL tube containing 20 mM of NaOH (200 µL). These tubes were set at 60 °C for 20 to 30 min. The NaOH was then aspirated and the disk was equilibrized in 200 µL of TE^−1^ buffer (0.1 mM EDTA, 10 mM Tris-HCl, pH 8.0). This buffer was then aspirated, and the disks were allowed to dry at ambient temperature until used to prime polymerase chain reaction (PCR) amplifications.

### 2.2. Genotyping of KRTAP20-1 Utilising PCR Amplification and SSCP Genotyping

PCR amplification was conducted utilising the PCR primers described in Gong et al. [16]. The reaction was undertaken in a 15 μL reaction volume that contained 2.5 mM Mg^2+^, 0.25 μM of primer, 150 μM of each dNTP, 0.5U Taq DNA polymerase (Qiagen, Hilden, Germany), 1× reaction buffer supplied with the enzyme, and the genomic DNA on a washed and equilibrated 1.2 mm TFN card disk. The amplifications were undertaken in S1000 thermal cyclers (Bio-Rad, Hercules, CA, USA) with a preliminary denaturation of 2 min at 94 °C, followed by 35 cycles of 94 °C for 30 s, 60 °C for 30 s, 72 °C for 30 s, and an ultimate extension/polishing step of 5 min at 72 °C.

PCR amplicon genotyping uses a single stranded conformation polymorphism (SSCP) analysis approach. In this method, a 0.7 μL portion of each amplicon was mixed with 7 μL of gel loading dye (0.025% xylene-cyanol, 0.025% bromophenol blue, 10 mM EDTA, and 98% formamide) and denatured at 90 °C for 5 min. The samples were then immersed in wet ice. They were immediately loaded onto 16 cm × 18 cm, 14% acrylamide: bisacrylamide (37.5:1; Bio-Rad) gels containing 1% glycerine. Protean II xi cells (Bio-Rad) were used with coolant circulating at 8 °C, and the gels were electrophoresed at 390 V in 0.5× TBE buffer for 20 h. Samples producing the DNA sequence variants identified previously in Gong et al. [16] were obtained from the authors and run in each gel as references to determine the variants present in each Tan sheep sample.

The gels were silver stained by being immersed in a solution of 0.5% acetic acid, 10% ethanol, and 0.2% silver nitrate for 10 min. They were then washed with distilled water and developed with a solution of 3% NaOH and 0.1% HCOH until dark staining bands materialized in the yellow backdrop of the gel.

### 2.3. Statistical Analyses

All statistical analyses were performed using Minitab version 16 (Minitab Inc., State College, PA, USA). First, phenotypic data were checked to confirm that they were normally distributed. Pearson correlation coefficients were then determined to test the strength of the correlation among MFD, FDSD, CVFD, and MFC. Next, General Linear Models (GLMs) were used to assess the effect of the absence or presence (coded as 0 or 1, respectively—a binomial GLM) of *KRTAP20-1* variants on the four fibre traits, but only for variants with frequencies greater than 5%. Variants present in only a small number of Tan lambs might bias the analyses if these lambs have an extreme phenotype. In these models, sex and sire were ascertained to affect all wool traits, so they were entered as fixed and random factors, respectively.

The model used to test the variant presence/absence effect was Y_ijkl_ = μ + V_i_ + Sex_j_ + Sire_k_ + e_ijkl_, where Y_ijkl_ is phenotype of the ijkl-th sheep (for either MFD, CVFD, FDSD, or MFC), μ = is the group raw mean for the trait, V_i_ is the effect of the i-th variant (presence and absence), Sex_j_ is the fixed effect of j-th sex, Sire_k_ is the random effect of kth sire, and e_ijkl_ is the random residual effect.

A trend towards association was recorded when 0.05 ≤ *p* < 0.1, and *p* < 0.05 was accepted as indicative of significance.

## 3. Results

### 3.1. Polymorphism of KRTAP20-1 in Tan Sheep

The PCR-SSCP typing system revealed banding patterns indicating seven DNA sequence variants of *KRTAP20-1* in the Tan sheep. These banding patterns were identified by matching them to the banding patterns previously identified by Gong et al. [16] for variants *A*, *B*, *D*, *E*, *F*, *G*, and *H* of *KRTAP20-1*. The haplotype sequences of all variants are detailed in Table 1. Variant *C* was not discovered in the 227 Tan sheep. The amplicon size of all these variants was 290 base pairs (bp), except for variant *H*, which contained a 12 bp deletion, resulting in it having a size of 278 bp.

Thirteen genotypes of these variant sequences were observed among the 227 lambs typed. The genotype frequencies are presented in Table 2. This distribution corresponds to variant frequencies of 74.7% (*A*), 4.0% (*B*), 4.6% (*D*), 4.4% (*E*), 0.7% (*F*), 11.5% (*G*), and 0.2% (*H*) for the Tan sheep samples analysed.

### 3.2. Associations between Variation in KRTAP20-1 and Wool Traits

Of the seven variants revealed in the 227 Tan lambs, *B* (*n* = 14 lambs carried this variant), *D* (*n* = 16), *E* (*n* = 20), *F* (*n* = 3), and *H* (*n* = 1) appeared at a frequency below 5%, and sheep with these variants were excluded from the presence versus absence models because of the small sample size and potential bias that may result. This left two variants: *A* (*n* = 200 lambs carried this variant) and *G* (*n* = 40) that were analysed to ascertain if they affected wool traits.

The models revealed that the *KRTAP20-1* variation influenced MFC in the fine wool fibres. Lambs with the *G* variant produced wool with higher MFC compared to those with the *A* variant (*p* = 0.017; Table 3). A trend of association with MFD was also detected for the fine wool. No other associations were observed for other traits, or for the heterotypic hair fibre.

Strong correlations (|r| > 0.7) were found between FDSD and CVFD in the Tan lamb wool fibres and the heterotypic hair fibre samples (Table 4). Moderate correlations (0.3 < |r| ≤ 0.7) were found between MFD and FDSD, between MFD and CVFD, and between MFD and MFC in the fine wool fibre samples, and between MFD and FDSD in the hair samples (Table 4). There were only weak or negligible correlations (|r| ≤ 0.3) between the other wool traits (Table 4).

## 4. Discussion

This study revealed variation in *KRTAP20-1* in Tan sheep in China, and the variation in the gene reflects that which has been observed in a cross of Merino and Southdown sheep in New Zealand [16]. Compared with the eight variants reported in the Merino × Southdown-cross sheep, only seven variants were found in the Chinese Tan sheep, although differences were observed in variant frequencies in the two populations, which is not surprising given the breed differences. The Merino × Southdown-cross sheep had variant frequencies of *A* (70.4%), *B* (6.7%), and *C* (12.9%), and the remaining variant (*D* to *H*) frequencies were less than 5%. In comparison, there was no occurrence of variant *C* in the Chinese Tan sheep, and less than 5% for *B*, *D*, *E*, *F*, and *H*, with variant *A* being very common (74.7%), followed by *G* (11.5%).

Comparison of the sequences of variant *A* and variant *G* revealed they differed by the SNP c.-17C/T, which is in the 5′ untranslated region (5′UTR), 17 bp upstream of the start codon. Lambs with the *G* variant (c.-17T) produced fine wool with a higher average curvature than those with the *A* variant. While the difference between *A* and *G* is not in identified coding sequence, the 5′UTR region proximal to coding sequences can contain regulatory elements, such as structural components involved in the regulation of mRNA stability, pre-mRNA splicing, and translation initiation, but also other ORFs, ribosome entry sites, and microRNA binding sites [17]. Sequence variation in this region may therefore influence either gene transcription or translation, thus resulting in the abundance of the protein.

The association detected in this study suggests that variation in *KRTAP20-1* affects wool fibre curvature. *KRTAP20-1* belongs to the HGT-*KRTAP* group, and the HGT-*KRTAPs* are thought to be preferentially expressed in orthocortical cells [18]. An altered abundance of KAP20-1 protein may affect the proportion or distribution of orthocortical cells within the cortex, or impact fibre matrix crosslinking with the KIFs, ultimately influencing wool fibre curvature. In this respect, reports have linked the abundance of HGT-KAP proteins with fibre curvature in Merino and Romney sheep wool [19,20]. Plowman et al. [21] reported higher levels of specific HGT-KAP proteins in Merino wool fibres (fine wool) compared to Bordaleiro (medium wool) and Churro (coarse wool) wool fibre, and, if a similar effect was seen with the two fibre types in the Chinese Tan sheep, then one might expect the content of the KAP20-1 protein to be lower in the heterotypic hair fibres. This might then explain why the association of *KRTAP20-1* variation and MFC was observed with fine wool fibres, but not with the heterotypic hair fibres.

With the fine wool fibres, the MFC and MFD were moderately negatively phenotypically correlated (−0.399, *p* < 0.001). This suggests that the trend towards association detected between variant *G* and fine wool fibre MFD may be because of the correlation with MFC, rather than being an independent effect. If the variation in *KRTAP20-1* was independently affecting MFD, then an association, or trend towards association, with FDSD might also have been expected, given that a stronger MFD–FDSD correlation (0.664, *p* < 0.001) was revealed, compared to the correlation of MFD and MFC.

Compared to the study of *KRTAP20-1* in Merino × Southdown-cross sheep [16], the Tan sheep studied here exhibited a slightly higher frequency of variant *A*. Gong et al. [16] revealed this variant to be associated with lower MFD in the Merino × Southdown-cross sheep, while no association was observed in this study (and the marginal mean MFD for the Tan sheep with variant *A* was higher). Additionally, the Tan sheep had a higher frequency of variant *G*, which was trending towards an association with lower MFD compared to variant *A* in fine wool. Variant *G* was rare (with a frequency of less than 5%) in the Merino × Southdown-cross sheep. Care should be taken in making comparisons, however, because the fine wool fibres of Tan sheep were much finer than the Merino × Southdown wool described by Gong et al. [16], with MFD in the range of 16-17 microns, compared to 19-21 microns.

Although *KRTAP20-2* belongs to the *KRTAP20* family gene, and *KRTAP20-2* is also associated with MFC [2], the genes *KRTAP6-3* and *KRTAP36-1* are the two closest known *KRTAPs* flanking *KRTAP20-1* on sheep chromosome 1. *KRTAP6-3* is located approximately 44 kb away from *KRTAP20-1* on one side [16], while *KRTAP36-1* is approximately 52 kb away from *KRTAP20-1* on the other side [22]. Variation in *KRTAP6-3* is associated with variation in MFD [23], while variation in *KRTAP36-1* is linked to increased prickle factor in wool [22]. Any traits associated with variation in *KRTAP20-1* may therefore be from the effect of the *KRTAP20-1* gene itself, or because of linkage to nearby *KRTAPs* that are variable. In either case, the possibility also exists that there are unidentified *KRTAP* genes in proximity to *KRTAP20-1*, given the clustering of the *KRTAPs*.

Further investigation of this part of the genome therefore appears to be warranted, both in Chinese Tan sheep and other breeds, especially if *KRTAP20-1* is to have the potential to be a genetic marker for enhancing fine wool fibre quality in the breeding of Chinese Tan sheep.

## Figures and Tables

**Table 1:** Ovine *KRTAP20-1* variants, accession numbers, and haplotypes.

Variant	Accession Number	Haplotype ^†^
KRTAP20-1*A	MH243552	[c.-17c; c.28G; c.59G; c.135A; c.153G; c.*24c]
KRTAP20-1*B	MH243553	[c.-17c; c.28G; c.59G; c.135A; c.153G; c.*24t]
KRTAP20-1*C	MH243554	[c.-17c; c.28G; c.59A; c.135A; c.153G; c.*24c]
KRTAP20-1*D	MH243555	[c.-17c; c.28G; c.59G; c.135G; c.153G; c.*24c]
KRTAP20-1*E	MH243556	[c.-17c; c.28A; c.59G; c.135A; c.153G; c.*24c]
KRTAP20-1*F	MH243557	[c.-17c; c.28G; c.59G; c.135A; c.153A; c.*24c]
KRTAP20-1*G	MH243558	[c.-17t; c.28G; c.59G; c.135A; c.153G; c.*24c]
KRTAP20-1*H	MH243559	[c.-17c; c.28G; c.59G; c.63_74del; c.135A; c.153A; c.*24c]

^†^ Lower case letters are non-coding DNA.

**Table 2:** Genotype frequencies in the 227 Tan sheep lambs.

Genotype	*AA*	*AB*	*AD*	*AE*	*AF*	*AG*	*AH*	*BB*	*BD*	*DD*	*DE*	*EG*	*GG*
Count	140	8	8	15	3	24	1	4	2	5	1	4	12
Frequency (%)	61.7	3.5	3.5	6.6	1.3	10.6	0.4	1.8	0.9	2.2	0.4	1.8	5.3

**Table 3:** Association of *KRTAP20-1* variants with four fibre diameter-related measurements from Tan sheep.

Wool Type	Trait ^1^	Variant ^2^	Mean ± SE ^3^		*p*
			Absent	Present	
Fine wool	MFD (μm)	*A*	16.2 ± 0.43	16.7 ± 0.19	0.290
		*G*	16.8 ± 0.20	16.3 ± 0.26	*0.065*
	FDSD (μm)	*A*	3.8 ± 0.31	4.2 ± 0.13	0.258
		*G*	4.2 ± 0.14	4.0 ± 0.19	0.190
	CVFD (%)	*A*	23.4 ± 1.46	24.9 ± 0.63	0.322
		*G*	25.0 ± 0.67	24.2 ± 0.89	0.414
	MFC (°/mm)	*A*	64.2 ± 2.91	64.3 ± 1.25	0.986
		*G*	63.0 ± 1.22	67.3 ± 1.70	**0.017**
Heterotypic wool	MFD (μm)	*A*	28.7 ± 0.88	29.6 ± 0.38	0.406
		*G*	29.6 ± 0.40	29.5 ± 0.54	0.888
	FDSD (μm)	*A*	7.7 ± 0.38	8.2 ± 0.17	0.138
		*G*	8.3 ± 0.18	8.0 ± 0.24	0.268
	CVFD (%)	*A*	26.7 ± 1.21	27.9 ± 0.52	0.321
		*G*	28.0 ± 0.55	27.1 ± 0.74	0.246
	MFC (°/mm)	*A*	46.0 ± 1.80	46.3 ± 0.78	0.884
		*G*	46.5 ± 0.82	45.8 ± 1.11	0.561

^1^ MFD—mean fibre diameter; FDSD—fibre diameter standard deviation; CVFD—coefficient of variation of fibre diameter; MFC—mean fibre curvature. ^2^ A total of 176 sheep are included in the model when variants that occurred at a frequency under 5% are excluded. Variant *A* is present in 164 sheep and absent in 12 sheep, and variant *G* is present in 36 sheep and absent in 140 sheep. ^3^ Predicted means and standard errors of those means derived from GLMs, with *p* < 0.05 being presented in bold and 0.05 ≤ *p* < 0.10 being italicized.

**Table 4:** Pearson correlation coefficients and significance levels for comparisons of the hair and wool fibre traits.

	Fine Wool				Heterotypic Wool		
Traits ^1^	MFD (μm)	FDSD (μm)	CVFD (%)		MFD (μm)	FDSD (μm)	CVFD (%)
FDSD (μm)	0.664 0.000				0.453 0.000		
CVFD (%)	0.362 0.000	**0.934** 0.000			−0.208 0.004	**0.772** 0.000	
MFC (°/mm)	−0.399 0.000	−0.278 0.000	−0.175 0.015		−0.016 0.820	0.121 0.092	0.151 0.035

^1^ MFD—mean fibre diameter; FDSD—fibre diameter standard deviation; CVFD—coefficient of variation of fibre diameter; MFC—mean fibre curvature. Correlations with |r| > 0.7 are in bold, and those with 0.3 < |r| ≤ 0.7 are underlined.

## Data Availability

The original data used in this paper are available upon request by contacting the corresponding author.
